# A systematic morphology study on the effect of high glucose on intervertebral disc endplate degeneration in mice

**DOI:** 10.1016/j.heliyon.2023.e13295

**Published:** 2023-01-31

**Authors:** Huilin Quan, Xiaoshuang Zuo, Yu Huan, Xuankang Wang, Zhou Yao, Chunmei Wang, Fang Ren, Hong Wang, Hongyan Qin, Xueyu Hu

**Affiliations:** a Department of Orthopedics, Xijing Hospital, Fourth Military Medical University, Xi’an, 710032 Shaanxi China; b Department of Neurosurgery, Xijing Hospital, Fourth Military Medical University, Xi’an, 710032 Shaanxi China; c Department of Radiology, Xijing Hospital, Fourth Military Medical University, Xi’an, 710032 Shaanxi China; d State Key Laboratory of Cancer Biology, Department of Medical Genetics and Developmental Biology, Fourth Military Medical University, Xi'an, 710032 Shaanxi China

**Keywords:** Diabetes, Endplate, Vascular invasion, Monocyte/macrophage infiltration, Inflammation

## Abstract

To explore the relationship between diabetes and intervertebral disc degeneration in mice and the associated underlying mechanism. Four-week-old male Kunming mice were used to model diabetes using a high-fat diet combined with streptozotocin injection. After 6 months, morphological and pathological changes in L4-L6 intervertebral discs were detected by magnetic resonance imaging, micro-CT and histological staining. Immunostaining of CD31, F4/80 and CD16/32 receptors was used to detect vascular invasion and inflammatory infiltration in endplates; the exact changes were then explored by the transmission electron microscopy. The nucleus pulposus of the control and the diabetic group had a clear boundary and regular shape without collapse, while endplate calcification and chondrocyte abnormality in the diabetic group were more obvious. Immunofluorescence confirmed that compared to control, expression levels of CD31 (vascular endothelial marker) and F4/80 (monocyte/macrophage marker) in the diabetic group were significantly increased (P < 0.05), with an elevated number of F4/80 (+)/CD16/32 (+) cells (P < 0.05). The morphology of endplates was observed by transmission electron microscopy, which showed monocytes/macrophage accumulation in the endplate of the diabetic group, accompanied by increased vascular density, collagen fiber distortion and chondrocyte abnormality. In a conclusion, diabetes promotes endplate degeneration with vascular invasion, monocyte/macrophage infiltration and inflammation in mice.

## 1 Introduction

Lumbar intervertebral disc degeneration is a common orthopedic degenerative disease and may be caused by many factors, including age, posture, instability, obesity and diabetes. It is worth noting that diabetes is an endocrine disease with a characteristic of abnormally high glucose. Diabetes, mostly prevalent worldwide, is predicted that it may affect 693 million people by 2045, with a tremendous increase in comparison with 2017. In addition of the harm of diabetes itself, the various complications of diabetes attract the attention of the academic community, especially the major type of diabetes, type II diabetes mellitus (T2DM) [1]. Recent studies have shown that diabetes, with particular reference to T2DM, can accelerate the process of intervertebral disc degeneration. Intervertebral disc degeneration is a prevalent existence in people with T2DM [2] and these patients are more vulnerable to disc degeneration [3]. Even though there are some biological factors related to T2DM such as advanced glycation end-products (AGEs), human islet amyloid polypeptide (hIAPP) and so on, aggrevating intervertebral disc degeneration, the specific mechanism remains unclear [[4], [5], [6], [7], [8]]. Endplate cartilage is primarily responsible for transport within the intervertebral discs [9], and its degeneration is an important factor in intervertebral disc degeneration [10]. It has been shown that high glucose can promote apoptosis of rat endplate chondrocytes via ROS accumulation, changes to the mitochondrial membrane potential, up-regulation of pro-apoptotic proteins and down-regulation of anti-apoptotic proteins. Jiang [11] showed that high glucose can promote endplate chondrocyte apoptosis in rats and that MALAT1, which encodes a non-coding mRNA, is an important regulatory gene. Inhibition of MALATA1 can reduce levels of phosphorylated p38 and total p38 and reduce cell apoptosis. However, our current understanding of the mechanism by which diabetes induces endplate degeneration is far from adequate, and most studies to date have focused on the cellular and genetic levels, without detailed evidence of morphology in vivo. Therefore, we established a model of diabetes in mice and examined the potential mechanism underlying endplate degeneration by means of magnetic resonance imaging, histological staining and transmission electron microscopy.

## 2 Materials and methods

### 2.1 Materials

4-week-old male Kunming mice were purchased from the Experimental Animal Center of Fourth Military Medical University and approved by the Animal Ethics Committee of Fourth Military Medical University (License number: IACUC-2020065). The animal experiment was complied with the ARRIVE guidelines and was performed in accordance with the National Institutes of Health guide for the care and use of Laboratory animals. 60% high-fat diet were purchased from Readydietech, China. Streptozocin (STZ) was purchased from Sigma, USA. Hematoxylin-Eosin (HE) was purchased from Solarbio, China. CD31 antibody was purchased from Abcam (28364, 1:100), USA. F4/80 antibody was purchased from Abcam (6640, 1:100), USA. CD16/32 antibody was purchased from BD Biosciences (553142, 1:50), USA. Fluorescence secondary antibody was purchased from Invitrogen, USA. IL-1β and TNF-α ELISA kits were purchased from Proteintech, USA.

### 2.2 Methods

#### 2.2.1 Diabetes models

4-week-old male Kunming mice were divided into 2 groups randomly. The diabetic group mice were fed with high-fat diet for 4 weeks, fasted for 12 h, and had an intraperitoneal injection with 70 mg/kg STZ solution (STZ dissolved in 0.1 mol/L citric acid buffer prepared by citric acid and sodium citrate) to induce a diabetes model. The mice were observed after injections. The tail vein blood of mice was collected once a week to measure random blood glucose for 6 months. The mice with blood glucose ≥16.7 mmol/L were selected as eligible model mice for subsequent experiments. Mice in the control group were fed normally [12,13]. And on the 7th day after the STZ injection, intraperitoneal glucose tolerance tests (ipGTT) were performed (1 g/kg body weight) [14]. Blood samples were taken from the mouse tail at 0, 0.5, 1, 2 and 3 h after fasting for 16 h [15].

#### 2.2.2 Magnetic resonance imaging (MRI)

The mice were examined by MRI, following 6 months of the model establishment. Anesthesia with pentobarbital sodium solution (0.6%, 0.01 ml/g), was used as intraperitoneal injection. After successful anesthesia, the mice were fixed in the supine position on the examination bed, and the tail was in the same line with the spine. After that, mice with stable breathing could be used for detection. The T2 weighted images were obtained after scanning, and the L4–L6 intervertebral discs were selected as the observation objects (the anterior superior iliac spine of mice was L6, and the upward segments were L4 and L5). The mean density of L4-L6 nucleus pulposus was calculated.

#### 2.2.3 Micro-CT

The lower lumbar spine (L4-L6) of mice were dissected when the MRI examination was done, fixed in 10% buffered formalin for 48 h and then examined by micro-CT (Locus_SP). Images were reconstructed and analyzed using MS-8. The scanner was set at a voltage of 80 kVp, a current of 80 μA, an exposure time of 3000 ms and a resolution of 7.9 μm to measure the endplates. Endplate volume was defined to the cartilage part covering the vertebrae. Two parameters, including bone mineral density (BMD) and bone volume/tissue volume (BV/TV) were used to analyze the endplates.

#### 2.2.4 Histological staining

The mice after micro-CT examination were sacrificed and L4-L6 segments were taken. These segments were fixed with 4% paraformaldehyde for 48 h, and cut into 5 μm tissue sections after decalcification. Then, these sections were dyed with hematoxylin for 2–20 min, washed with distilled water, differentiated in differentiation medium for 3 min and washed with distilled water twice again. The slides were dipped in Eosin dye solution for 30s-2 min and washed with distilled water for 2–3s. The results were observed with a microscope.

#### 2.2.5 Immunofluorescence staining

The tissue sections were subjected to antigen retrieval, and PBS with 0.3% Triton X-100 was added for 15 min. The sections were blocked for 1 h at room temperature, using PBS with 3% BSA. Then the blocking solution was removed. The diluted primary antibody was added and the slides were placed in a wet box at 4 °C overnight. After washed with PBS, diluted secondary antibody was added and incubated at room temperature for 2 h. Then the nuclei were stained with DAPI and washed with PBS. Finally, the anti-quenchant was added and photographed with a fluorescence microscope (Olympus, Japan).

#### 2.2.6 Transmission electron microscopy (TEM)

After the mouse endplates were taken out according to the method above, they were fixed in 2.5% glutaraldehyde for 2 h and then decalcified. After decalcification and embedding, 60 nm ultra-thin slice were prepared. The results were photographed with a transmission electron microscopy.

#### 2.2.7 ELISA

The concentrations of IL-1β and TNF-α in the endplates were detected by ELISA kits according to the instructions, respectively. Endplate tissues were collected as samples and were added to the well with standards. After incubation and washing with wash buffer, detection and HRP-conjugated antibody solution were added for 40 min s and wash well. Then, TMB substrate solution was added to incubate for 20 min s and stop solution were used finally. Read the absorbance on a microplate reader at a wavelength of 450 nm.

#### 2.2.8 Statistical analysis

Graphpad Prism 8.0 was used to analyze data and graphics, and the data were expressed as‾x± SEM in each group. Independent *t*-test was used for comparison between groups. ns：no significance, *p < 0.05, **p < 0.01, ***p < 0.001, ****p < 0.0001.

## 3 Results

### 3.1 High glucose promotes intervertebral disc degeneration in mice

**Fig. 1:**
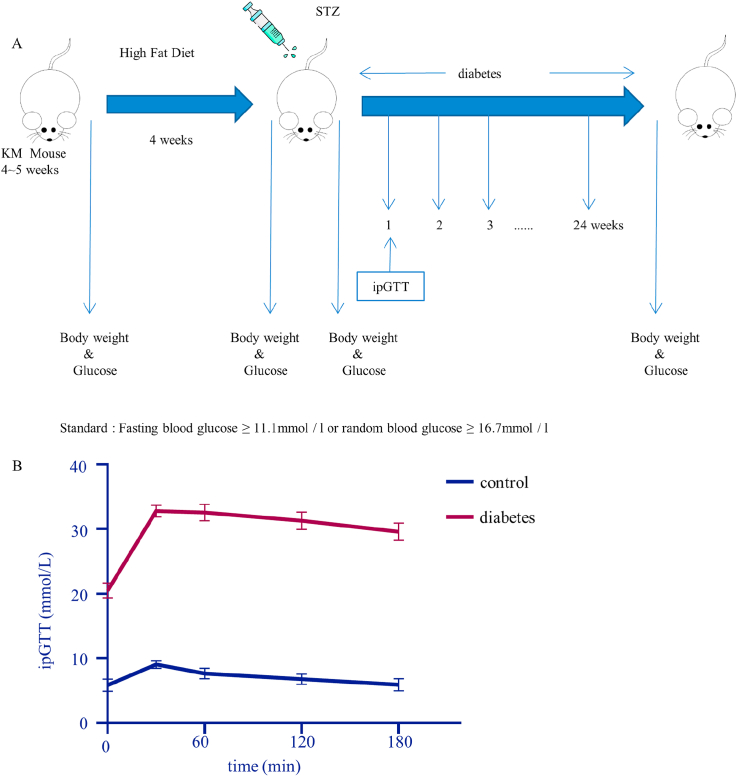
The diabetes model was established. A. Schematic diagram of T2DM model in mice. B. The ipGTT results in the control and diabetic group.

**Fig. 2:**
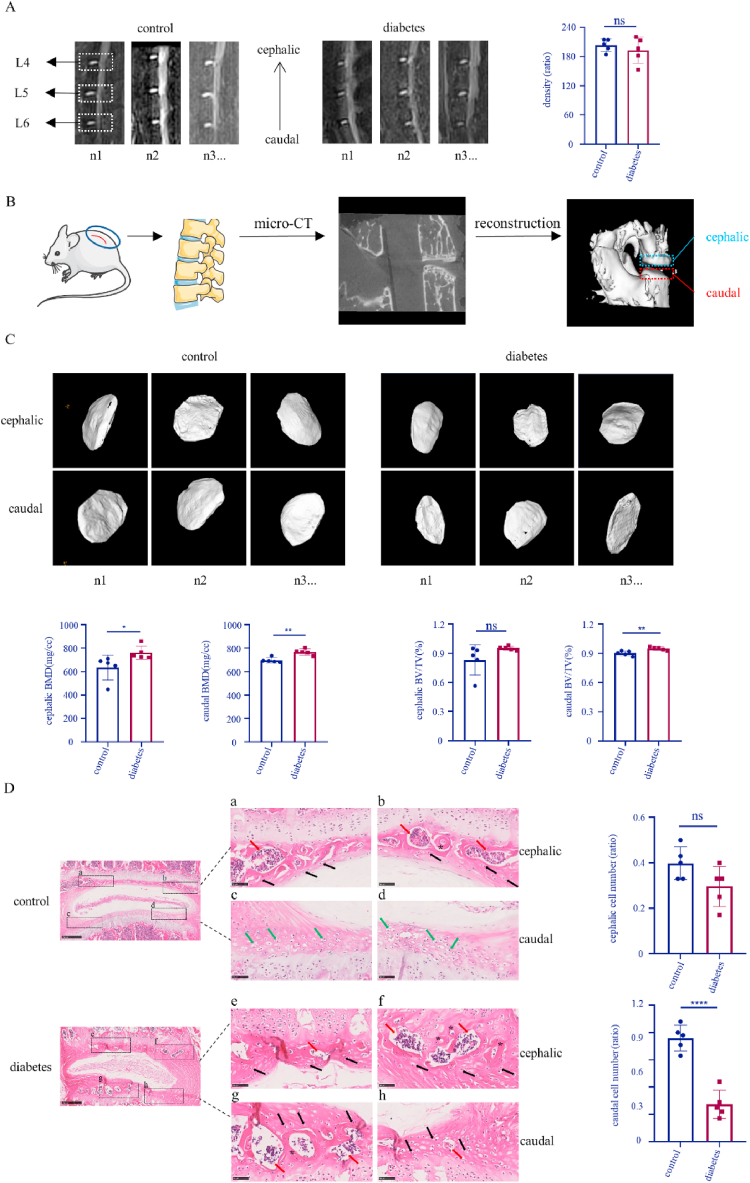
Diabetes increases endplate degeneration in mice. A: MRI of mouse intervertebral discs; B: The flow chart of mouse endplate micro-CT examination and reconstruction; C: Representative images of endplate micro-CT and quantitative analysis of the BMD and BV/TV of cephalic and caudal endplates based on the micro-CT images; D: H&E staining of mouse intervertebral discs and quantitative analysis of normal chondrocyte number. Red arrows: calcification; Black arrows: abnormal chondrocytes; Green arrows: normal chondrocytes; *: blood vessels. *p < 0.05, **p < 0.01, ****p < 0.0001 (n = 5). Scale bars are labeled in the figure. (For interpretation of the references to colour in this figure legend, the reader is referred to the Web version of this article.)

First, a diabetes model was established according to the method shown in Fig. 1A. Diabetic mice were prepared using a high-fat diet combined with intraperitoneal STZ injection. According to the blood glucose (Table 1, Table 2) and ipGTT (Fig. 1B) results, random blood glucose in the diabetic group was 16.7 mmol/L or more, with an abnormal ability of regulating blood glucose level. These mice were included in consequent experiments. In order to examine whether high glucose has an effect on intervertebral disc degeneration in mice, MRI was performed. The results (Fig. 2A) show that the nucleus pulposus of L4-L6 segments in the control group and diabetic group had a regular shape and were homogeneously white. There was a clear boundary between the anulus fibrosus and nucleus pulposus. Disc height was normal with no obvious extrusion or collapse. According to calculating the nucleus pulposus density, the difference between the control group and the diabetes group was non-significant (control vs diabetes: 203.4 vs 193.4; *p* = 0.4736). Endplates did not show any roughness, ambiguity, change in thickness change or signal intensity abnormality. However, based on the micro-CT results, the BMD (control vs diabetes: 697.0 vs 767.2; *p* = 0.0017) and BV/TV (control vs diabetes: 0.9001 vs 0.9477; *p* = 0.0081) of caudal endplates in the diabetic group were higher than caudal endplates in the control group. With regard to cephalic endplates, the BMD (control vs diabetes: 634.5 vs 761.6; *p* = 0.0475) in the diabetic group also increased, but the BV/TV (control vs diabetes: 0.8298 vs 0.9520; *p* = 0.1207) didn’t have an obvious fluctuation (Fig. 2B and C).

**Table 1:** Changes in body weight of diabetic and control mice after STZ injection. Means ± SEM. n = 10.

Body weight (g)								
0w		1w	4w	8w	12w	16w	20w	24w
control	46.86 ± 0.95	52.23 ± 1.02	55.11 ± 1.30	56.66 ± 1.03	57.51 ± 1.07	58.06 ± 1.04	58.62 ± 0.91	58.97 ± 0.91
diabetes	48.60 ± 1.28	48.92 ± 1.23	42.91 ± 0.61	42.12 ± 0.66	41.22 ± 0.56	40.64 ± 0.43	40.46 ± 0.38	40.33 ± 0.37

**Table 2:** Changes in blood glucose of diabetic and control mice after STZ injection. Means ± SEM. n = 10.

Blood glucose (mmol/L)								
0w		1w	4w	8w	12w	16w	20w	24w
control	7.49 ± 0.61	9.68 ± 0.88	9.29 ± 0.36	9.45 ± 0.26	9.25 ± 0.24	9.35 ± 0.23	9.45 ± 0.20	9.37 ± 0.20
diabetes	7.95 ± 0.34	27.54 ± 2.27	33.07 ± 0.22	33.19 ± 0.10	32.45 ± 0.48	32.75 ± 0.52	32.46 ± 0.48	33.15 ± 0.14

In order to more closely examine mouse intervertebral discs, histological staining was performed. The results (Fig. 2D) show that intervertebral disc height was normal, with no significant collapse in the control and diabetic group, consistent with our MRI findings. While the control group showed some calcification of the cephalic endplate and no calcification of the caudal endplate, the diabetic group had extensive calcification of both the cephalic and caudal endplate. It could be observed that the boundary between endplate and anulus fibrosus was rather clear in control group other than diabetic group. In the control group, there existed some vessels in the cephalic endplate other than the caudal endplate, in comparison with numerous vessels in both endplates of diabetes group. Meanwhile, inflammatory cells such as macrophages were besieged by these vessels. In addition, chondrocytes in the caudal endplate of the control group had normal morphology and were arranged back-to-back, while chondrocytes in the caudal endplate of the diabetic group appeared shriveled and the normal chondrocyte number was decreased (control vs diabetes: 0.7600 vs 0.2740; *p* < 0.0001). These variations in the cephalic endplate of both groups were with no obvious difference (control vs diabetes: 0.3980 vs 0.2960; *p* = 0.0800).

### 3.2 Diabetes contributes to the growth of blood vessels in the endplate

**Fig. 3:**
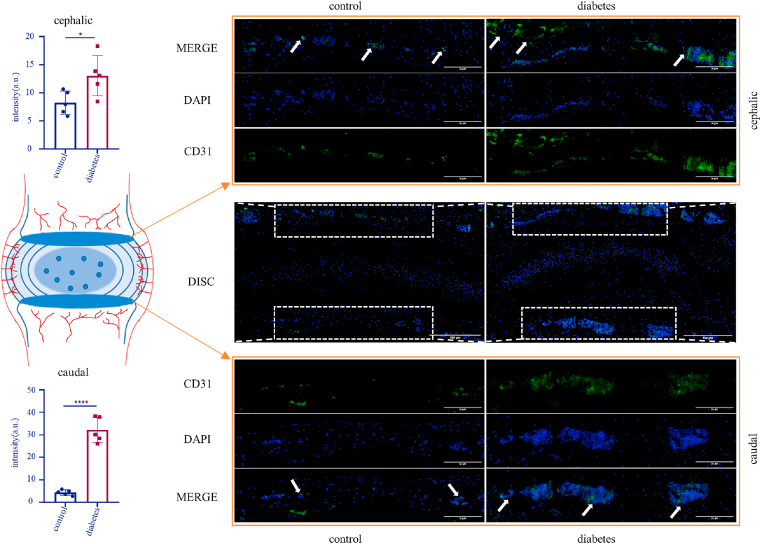
Immunofluorescence staining of CD31 in mouse intervertebral discs and quantitative analysis of the intensity of CD31(+) area. Arrow: CD31 (+) vascular endothelium. *p < 0.05, ****p < 0.0001 (n = 5). Scale bars are labeled in the figure.

Vascular invasion and inflammation are a sign of endplate degeneration [16,17]. Therefore, immunostaining of CD31 (a vascular endothelial marker) was performed on mouse disc slices [[18], [19], [20]]. The results showed high expression of CD31 in the cephalic endplate in the control group and diabetic group, indicating that peripheral vascular invasion occurred in both groups. But the invasion in the diabetic group was severer (control vs diabetes: 8.231 vs 13.04; *p* = 0.0320). Compared to control, the expression of CD31 in the caudal endplate of diabetic mice was obviously up-regulated (control vs diabetes: 4.386 vs 32.08; *p* < 0.0001), suggesting that high glucose can promote the invasion of peripheral blood vessels into the caudal endplate of mice (Fig. 3).

### 3.3 Diabetes facilitates monocyte/macrophage infiltration

**Fig. 4:**
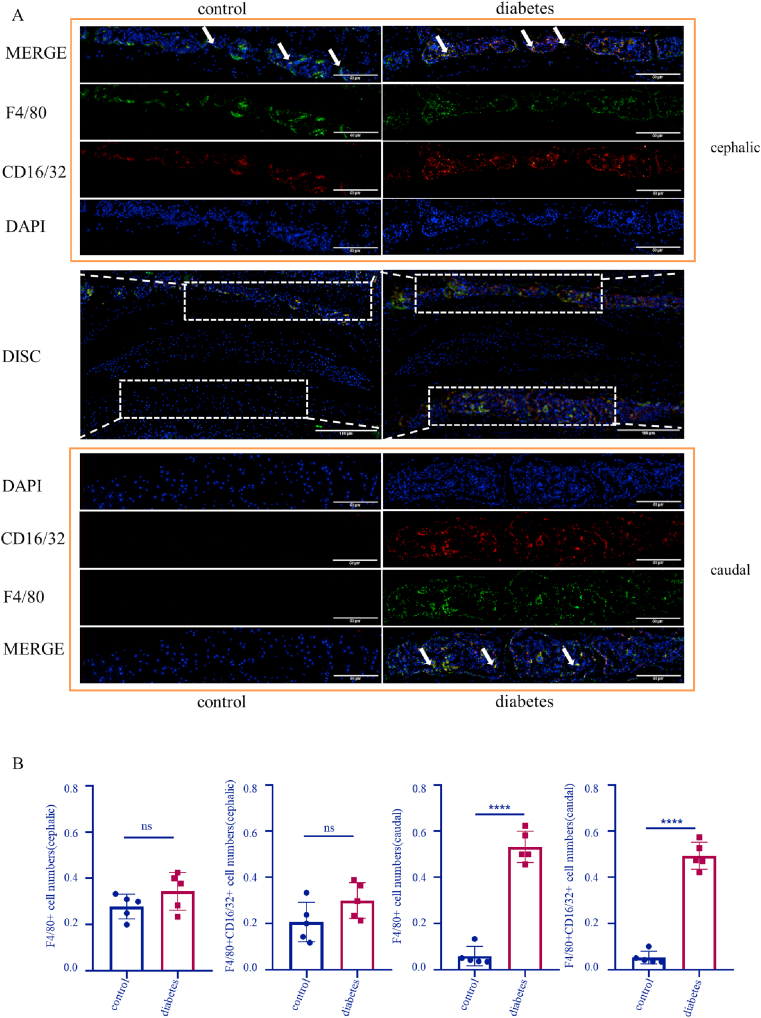
High glucose promotes monocyte/macrophage infiltration and expression of inflammatory factors. A: Immunofluorescence co-staining of F4/80 and CD16/32 in mouse intervertebral discs. Arrow: F4/80 (+)/CD16/32 (+) monocytes/macrophages. B: Analysis of F4/80 (+) cells and F4/80 (+)/CD16/32 (+) cells in endplates. ****p < 0.0001 (n = 5). Scale bars are labeled in the figure.

To confirm the monocytes/macrophages existence and determine whether high glucose promotes their pro-inflammatory effect, F4/80 (monocyte/macrophage marker) and CD16/32 (monocyte/macrophage marker) co-immunostaining was used. In the cephalic part, the results showed no significant difference in the number of F4/80 (+) cells (control vs diabetes: 0.2786 vs 0.0.3440; *p* = 0.1723) and F4/80 (+)/CD16/32 (+) cells (control vs diabetes: 0.2064 vs 0.2989; *p* = 0.1093) between the control group and the diabetic group. While in the caudal part, the number of F4/80 (+) cells (control vs diabetes: 0.059 vs 0.5324; *p* < 0.0001) and F4/80 (+)/CD16/32 (+) cells (control vs diabetes: 0.0524 vs 0.493; *p* < 0.0001) in the diabetes group were significantly higher than in the control group (Fig. 4A and B). In addition, expression levels of IL-1β and TNF-α in the endplate were assessed, and it was found that levels of IL-1β (control vs diabetes: 8.158 vs 15.13; *p* < 0.0001) and TNF-α (control vs diabetes: 104.6 vs 131.7; *p* < 0.0001) in the diabetic group were increased to various degrees (Supplementary figure). Thus, inflammatory monocyte/macrophage infiltration and inflammatory response can appear more commonly in the endplates of diabetic mice.

### 3.4 Inflammation and vascular invasion induced by diabetes can be confirmed at the ultrastructural level

**Fig. 5:**
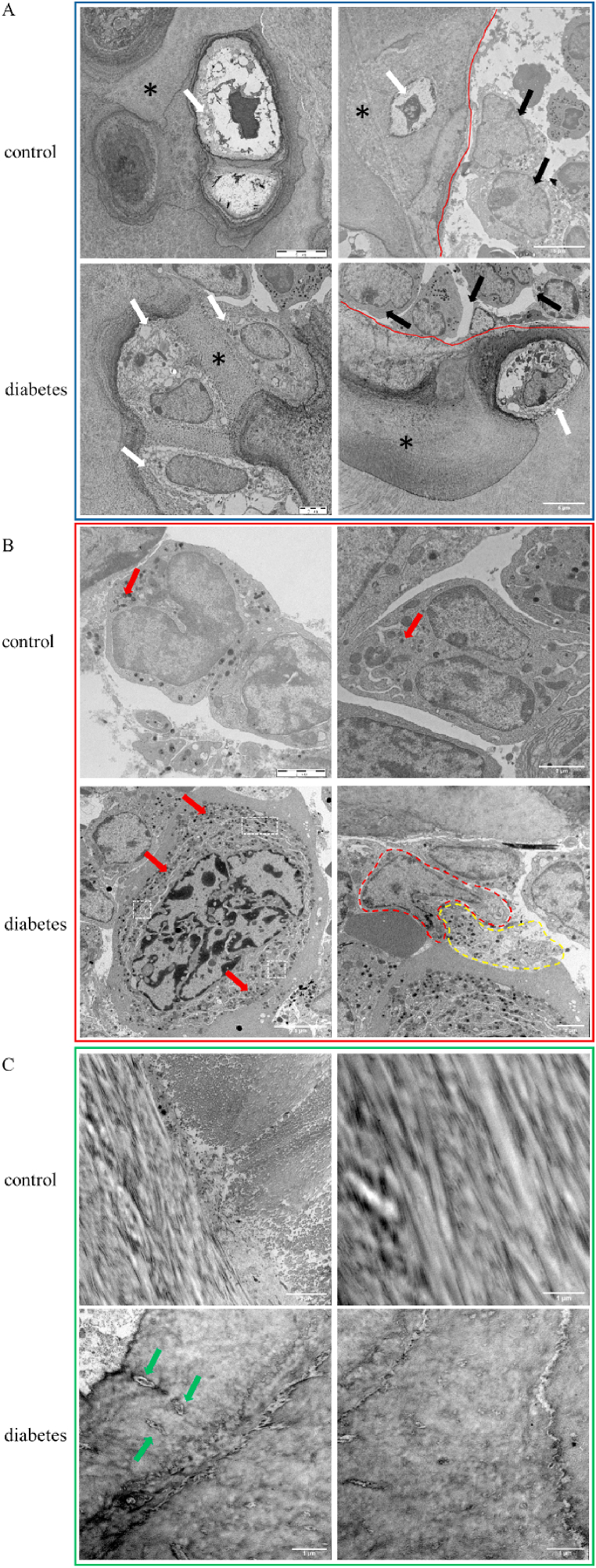
TEM detection of mouse endplates. A. Representative TEM images of mice in the control and diabetes group (chondrocytes). White arrow: endplate chondrocyte; *: extracellular matrix; Red line: vascular endothelium; Black arrow: monocyte/macrophage. B. Representative TEM images of mice in the control and diabetes group (monocytes/macrophages). Red arrow: lysosome; Red dotted area: monocytes/macrophages; Yellow dotted area: extracellular waste/debris; White dotted area: organelles compartments separated by endoplasmic reticulum. C. Representative TEM images of mice in the control and diabetes group (collagen fibers). Green arrow: fibroblast. Scale bars are labeled in the figure. (For interpretation of the references to colour in this figure legend, the reader is referred to the Web version of this article.)

To examine the ultrastructure of the endplate, TEM was performed. As shown in Fig. 5A, the chondrocyte nucleus in the control group had a regular shape and affluent cytoplasm. But the diabetic chondrocyte nucleus was in a senescent condition, and it was observed abnormal mitochondria and amounts of vacuolus in cytoplasm which pushed the nucleus to one side of the chondrocyte. In addition, the ability of extracellular matrix secretion in the control group was stronger than the diabetic group where the secreted extracellular matrix mixed with peripheral collagen, showing calcification. While vascular endothelial cells and monocytes/macrophages were found in both groups, the organelles of monocytes/macrophages in the diabetic group were more active. These cells can endocytose extracellular waste or debris, forming “compartment” organelles separated by the injured endoplasmic reticulum, which can then form autophagic bodies that may contribute to pathology (Fig. 5B). Besides, the results showed (Fig. 5C) that collagen fibers of endplates in the control group were regular, straight and continuous, with a coarse morphology, forming striations. However, collagen fibers of endplates in the diabetic group were irregular, discontinuous and had a tendency to crack, with some destroyed fibroblasts.

## 4 Discussion

Diabetes is a global health problem caused by a lack of insulin or insulin resistance [[21], [22], [23], [24]]. The number of patients worldwide suffering from diabetes is expected to reach more than 600 million by 2040 [21,25]. T2DM is the most common type of the disease, accounting for 85–90% [21,22,26]. T2DM is associated with bone and cartilage conditions, increasing the risk of fracture, which can lead to spinal canal stenosis and decrease the height of intervertebral discs [[27], [28], [29]]. In China and Japan, studies have shown a strong correlation between diabetes and lumbar disc degeneration [30]. People with long-term T2DM or poor blood glucose management are prone to having a more severe lumbar disc degeneration [31]. The endplate of the intervertebral disc is the main channel for nutrient exchange and plays an important role in the pathogenesis of intervertebral disc degeneration [9,32]. It has been shown that endplate calcification, mitochondrial dysfunction of endplate chondrocytes and imbalance of extracellular matrix synthesis are key causes of endplate degeneration [[33], [34], [35], [36], [37], [38]]. A study by Won shows that high glucose can induce ROS accumulation, mitochondrial damage and apoptosis in rat endplate chondrocytes [39]. However, most studies on high glucose and endplate degeneration are done at the cellular level, and studies in vivo are not sufficient to draw mechanistic conclusions. This study provides novel in vivo morphological evidence of the relationship between high glucose and endplate degeneration in mice.

In this study, a high-fat diet combined with STZ injection was used to induce diabetes in mice to study the effects of high glucose on endplates. First, L4-L6 intervertebral discs of diabetic and normal mice were scanned by MRI (anterior superior iliac spine: L6). The results showed no significant difference in these segments between the two groups; the nucleus pulposus showed a high signal intensity and clear boundary with normal morphology, other than some collapse. This indicated that in the diabetic and the control group, intervertebral disc degeneration was not obvious using MRI. Because MRI cannot be used to directly observe changes within the endplate or evaluate degeneration state, L4-L6 segments were examined by micro-CT. Using the technique, we found there was an increased bone tissue content in the diabetic group, implying a calcification existence. And H&E results showed significant endplate degeneration in the diabetic group, which manifested as cartilage calcification, ambiguous boundary, vessel invasion, inflammatory cell infiltration and abnormal shape of chondrocytes which resembled the effects of diabetes on chondrocytes in articular cartilage [40]. These performances could be regarded as biomarkers of endplate degeneration and provide a further proof of imaging results [[41], [42], [43], [44]]. According to Melgoza's study, the endplate in diabetic group had a severer degeneration [45]. In order to analyze the degree of endplate degeneration, we took a detection of blood vessel growth and immunostaining of CD31 was performed on endplates. We found higher expression of CD31 in the endplates of diabetic mice, indicating that diabetes can promote invasion of peripheral blood vessels into the endplate. The results of F4/80 and CD16/32 co-staining confirmed that, in addition to vascular invasion, there was infiltration of pro-inflammatory monocyte/macrophage infiltration into the endplates of diabetic mice. The intervertebral disc is the largest avascular tissue in the body and its degeneration is associated with inflammatory cell infiltration and angiogenesis. Healthy intervertebral discs are protected by physical and molecular barriers, limiting their contact with immunocytes. However, when degeneration occurs, the protective barrier is broken and immunocytes can infiltrate the disc [46]. Several studies have shown that intervertebral disc degeneration is accompanied by vascular growth and endplate vascularization. It is known that vascular endothelial growth factor (VEGF) is highly expressed in degenerative discs and is related to disease progression. Studies in different species (e. g. humans, mice, rats) have shown that CD31 staining is positive in degenerative intervertebral discs [16,[47], [48], [49]]. These findings are consistent with our results, which show that disc degeneration complicated by diabetes exhibits vascular invasion. It has previously been reported that macrophages in degenerative intervertebral discs can be polarized to a proinflammatory phenotype, participating in angiogenesis and infiltrating into the nucleus pulposus to accelerate intervertebral disc degeneration [16,50,51]. Similar results were found in this study, which suggests that monocyte/macrophage infiltration in the endplate of diabetic mice plays a role in promoting inflammation.

Finally, since histological and immunostaining staining allow only rough observation of endplate changes, the precise degeneration state could not be detected. We therefore applied TEM to examine the ultrastructure. TEM results showed that diabetes had a destructive effect on cellular morphology. At the same time, diabetes promoted chondrocyte degeneration, inhibited normal secretion of extracellular matrix and accelerated calcification. While immune activation was observed in both groups, seen as vascular endothelial and monocyte/macrophage infiltration, immune activation in the diabetic group was higher. These results suggest that diabetes can cause inflammation in endplates of mice, leading to endplate degeneration. Likely, in articular cartilage, T2DM could result in nucleus atrophy, mitochondrial abnormality and irregular tidemarks separated the calcification zone from non-calcification zone [52]. It also has been manifested that inflammation could jeopardize collagen in chondrocytes [53]. Cui discovered that cartilage inflammation could cause collagen fibers chaotic via TEM, as same as our results. But the collagen fibers got bigger and thicker which showed an opposite trend [54]. According to these studies, we could know diabetes and inflammation could indeed do harm to chondrocyte and induce cartilage degeneration. Yet the relationship between diabetes and inflammation remains us to investigate more.

Lumbar disc degeneration is accompanied by many pathological changes, in which the primary modic change is the lesion of the endplate and bone marrow, which can be detected by MRI. Based on MRI signal intensity, endplate degeneration can be divided into three modic types: type I (fibrous tissue); II (adipose tissue); and III (sclerotic tissue) [[55], [56], [57]]. In terms of disease progression, the earliest modic change is edema. With the continuous stimulation of inflammatory, physical and chemical factors, endplate degeneration becomes more serious and develops to modic change type III, which involves endplate and subchondral bone calcification. This indicates that in the process of endplate degeneration, inflammation promotes endplate calcification, affects the nutritional transport function of the endplate in intervertebral discs and accelerates intervertebral disc degeneration [58]. One prospective cross-sectional study showed that the course and severity of diabetes were closely related to modic change [59]. Here, by establishing a mouse model of diabetes, we found substantial vascular invasion, monocyte/macrophage infiltration and high expression of inflammatory factors in diabetes-induced endplate degeneration. This explains the effect of high glucose on endplates from a morphological perspective in vivo. However, there existed some limitations in our study. First of all, we just explored the effect of high glucose on endplate degeneration preliminarily. The exact mechanism of endplate inflammation and degeneration caused by diabetes should have sufficient research. Moreover, the type of diabetes could be divided by a more disciplined standard and the experiments could be carried out from a multiple perspective with endocrinology such as biochemical analysis, insulin resistance and so on. Whereas there were these restrictions existing, our research did lay a foundation for future studies on the mechanism of diabetes and intervertebral disc degeneration.

## Author contribution statement

Huilin Quan: Conceived and designed the experiments; Performed the experiments; Analyzed and interpreted the data; Contributed reagents, materials, analysis tools or data; Wrote the paper.

Xiaoshuang Zuo, Yu Huan: Performed the experiments, Analyzed and interpreted the data.

Xuankang Wang, Zhou Yao, Chunmei Wang, Fang Ren, Hong Wang: Contributed reagents, materials, analysis tools or data.

Hongyan Qin, Xueyu Hu: Conceived and designed the experiments.

## Funding statement

Dr Xueyu Hu was supported by National Natural Science Foundation of China [81572151]; Shaanxi Provincial Key Research and Development Project [2021ZDLSF02-10]; Fourth Military Medical University [2018RCFC02 and XJZT21L01].

Prof Hongyan Qin was supported by Shaanxi Provincial Key Research and Development Project [2020ZDLSF03-05].

## Data availability statement

Data included in article/supplementary material/referenced in article.

## Declaration of interest’s statement

The authors declare no conflict of interest.
