# Supplementary figures and images for: A systematic morphology study on the effect of high glucose on intervertebral disc endplate degeneration in mice
Source: Heliyon. 2023 Jan 31;9(2):e13295. doi: 10.1016/j.heliyon.2023.e13295 (PMC9932476; doi:10.1016/j.heliyon.2023.e13295)

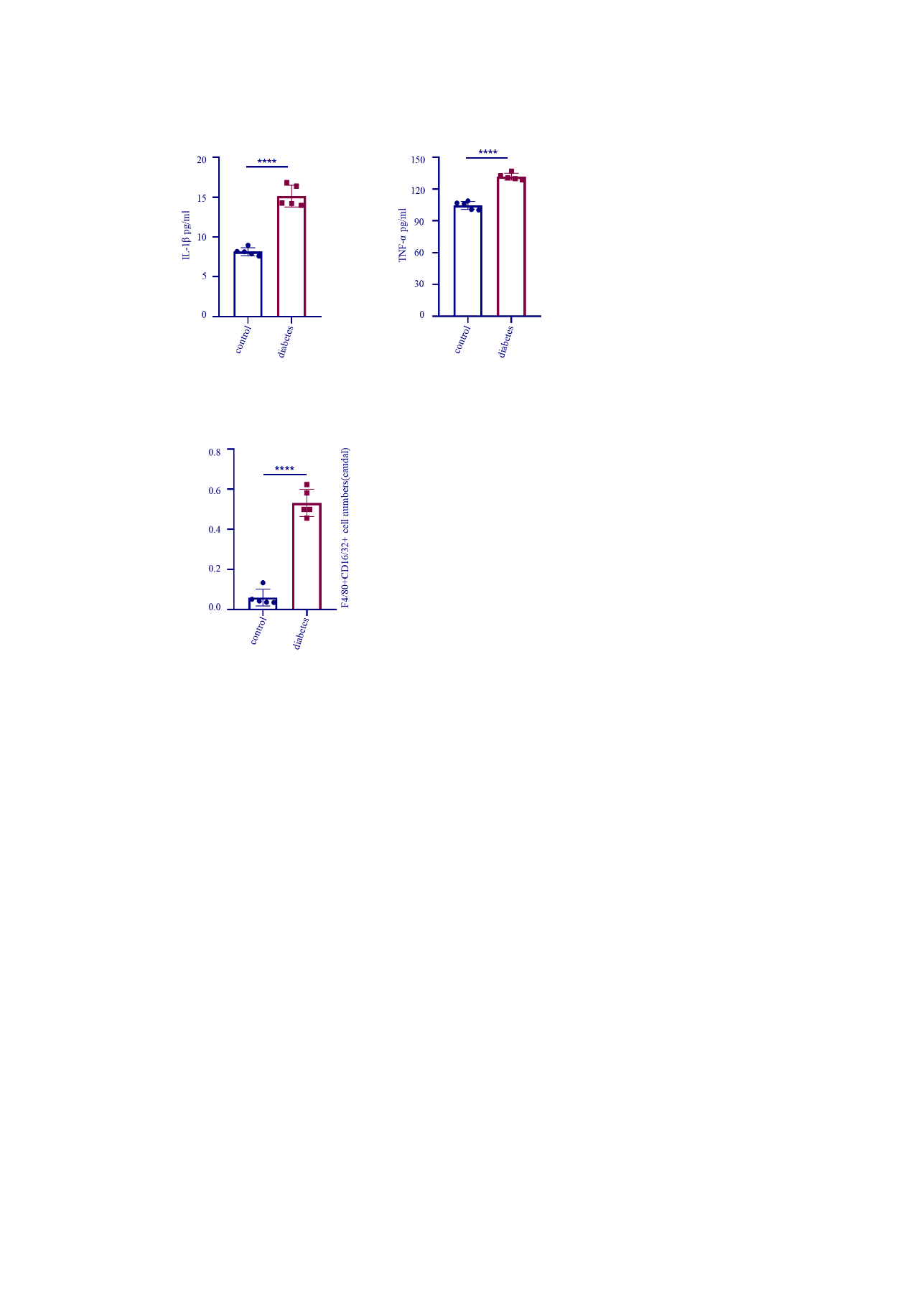


Analysis of IL-1β and TNF-α of ELISA detection in endplates. ****p<0.0001 (n = 5).

Supplement: Multimedia component 1 [file mmc1.docx]
